# Astrocyte - neuron lactate shuttle may boost more ATP supply to the neuron under hypoxic conditions - *in silico *study supported by *in vitro *expression data

**DOI:** 10.1186/1752-0509-5-162

**Published:** 2011-10-13

**Authors:** Seda Genc, Isil A Kurnaz, Mustafa Ozilgen

**Affiliations:** 1 Chemical Engineering Department, Yeditepe University, Istanbul, Turkey; 2 Genetics and Bioengineering Department, Yeditepe University, Istanbul, Turkey

## Abstract

**Background:**

Neuro-glial interactions are important for normal functioning of the brain as well as brain energy metabolism. There are two major working models - in the classical view, both neurons and astrocytes can utilize glucose as the energy source through oxidative metabolism, whereas in the astrocyte-neuron lactate shuttle hypothesis (ANLSH) it is the astrocyte which can consume glucose through anaerobic glycolysis to pyruvate and then to lactate, and this lactate is secreted to the extracellular space to be taken up by the neuron for further oxidative degradation.

**Results:**

In this computational study, we have included hypoxia-induced genetic regulation of these enzymes and transporters, and analyzed whether the ANLSH model can provide an advantage to either cell type in terms of supplying the energy demand. We have based this module on our own experimental analysis of hypoxia-dependent regulation of transcription of key metabolic enzymes. Using this experimentation-supported *in silico *modeling, we show that under both normoxic and hypoxic conditions in a given time period ANLSH model does indeed provide the neuron with more ATP than in the classical view.

**Conclusions:**

Although the ANLSH is energetically more favorable for the neuron, it is not the case for the astrocyte in the long term. Considering the fact that astrocytes are more resilient to hypoxia, we would propose that there is likely a switch between the two models, based on the energy demand of the neuron, so as to maintain the survival of the neuron under hypoxic or glucose-and-oxygen-deprived conditions.

## Background

Central and peripheral nervous system are composed of glia (astrocytes, oligodentrocytes and microglia) and neurons. Glia constitute 90% of the human brain cells; brain constitute up to 2% of total body weight, and consume about 20% of total body oxygen in the resting state. Reduction in the amount of oxygen in the blood (hypoxia) lead to intracellular regulation changes in astrocytes and neurons [1-3]. Glucose is usually considered the only carbon source for cerebral energy metabolism. Only about 1% of the total body glycogen is in the brain and it cannot be used as carbohydrate reserve in the brain cells [4,5]. Reducing the amount of glucose taken from blood to the brain leads to slow down of respiration and cerebral functions. Brain tissues are more sensitive to hypoglycemia when compared to the other organs. Glucose is taken to the brain cells from blood and catabolized to pyruvate and lactate in the cytoplasm, while oxidative respiration occurs in mitochondria. In recent years evidence implied that this compartmentalization may not be restricted to cytoplasm and mitochondrion only, but may also extend to the cellular level. Recently proposed Astrocyte-Neuron Lactate Shuttle Hypothesis (ANLSH) suggests that the glial glucose metabolism is almost completely anaerobic, and that the generated lactate which is released is transferred to neurons [4,6]. Recent studies have shown that the exogenous labeled lactate is a major substrate for oxidative metabolism in C6 neuronal cell lines [7] and neurons are capable of utilizing glucose in addition to lactate, down to CO_2_, whereas astroglial cells mainly metabolize glucose to lactate and released into the medium [8]. It was further shown that neurons cannot increase their rate of glycolysis whereas astrocytes can, simply because they lack a crucial glycolysis-promoting enzyme phosphofructokinase/fructose bisphosphatase, isoform 3 (PFKFB3) and glucose is utilized mostly through the pentose phosphate pathway generating glutathione and coping with oxidative stress, thus suggesting that glucose serves more as a survival factor than an energy source in neurons [9].

There is, in fact, other shuttle systems operating in the organisms - in the bee retina, for example, glucose is metabolized exclusively in the glia, and mitochondria are found exclusively in neurons [10]. In this system, glia were found to supply alanine to the neurons, and neurons return ammonium to the glia, suggesting a neuron-glia alanine-ammonium shuttle, and this study further implies lactate as a potential fuel supplied from the glia to the neuron [10]. Interestingly, enzymes that would be crucial to this shuttle, such as LDH, were shown to be regulated in a sleep-dependent manner: one of the many functions of sleep is supposed to be replenishing the energy stores in the brain; molecules that are potentially involved in regulating the lactate shuttle, such as LDH and GLUT1 in astrocytes, were shown to be activated during sleep deprivation, and similarly lactate shuttle was increased in wakefulness [11].

Lactate is a metabolite used also in hypoxia and normoxia in addition to anoxia, and lactate shuttle can be found in a variety of tissues including muscle, where there is a net flow of lactate from muscle to the blood, which is then recovered from the blood by the resting muscle cell and removed from the system by oxidation [12]. In the brain, lactate was reported to be an immediate energy source upon hypoxia; heart muscle is also an active consumer of lactate, and in muscle tissue lactate can also be taken up by the mitochondria by mitochondrial MCT transporters to be converted into pyruvate and consumed in the citric acid cycle [12]. Although not directly related to the ANLSH, there is evidence that monocarboxylates can act as rich energy sources for cells: cleavage-stage embryos, for example, initially require pyruvate but they switch to glucose as the preferred energy source as the embryo develops into a morula [13]. Lactate and pyruvate transport occurs via MCT transporters in the embryo, and blastocysts actually demonstrate higher affinity to lactate than zygotes [13]. As for neuronal cells, exogenous ^13^C-labeled lactate was shown to be a major substrate for oxidative metabolism in C6 cell lines, and hypoxic conditions were found to accumulate lactate as a rich energy source [7].

Neurons and astrocytes both express glucose transporters (GLUTs), lactate transporters (monocarboxylate transporters, MCTs), and lactate dehydrogenases (LDHs), however the different isoforms expressed by neurons or astrocytes seem to support the ANLSH model [14,15]. MCTs transport monocarboxylates such as pyruvate and lactate across plasma membrane or even mitochondrial membranes as in the case of MCT1 or MCT2 [16]; MCT1 is mostly ubiquitous, while MCT4 is mostly found in muscle cells or other metabolically active cells including tumors, while MCT2 is mostly found in kidney, neurons and sperm tails where rapid uptake of low concentration substrates is required [1]. MCT1, present in astrocytes, is known to be involved in preferential release of lactate, whereas MCT2, present in neurons, has been implied in the consumption of lactate. In a different study using HeLa and COS cells, it was shown that MCT4, but not MCT1, was upregulated by HIF-1a in hypoxia. In adipocytes, hypoxia was seen to upregulate MCT1 and MCT4 message, while decreasing MCT2 expression [3].

Neurons and astrocytes also express different glucose transporter isoforms - GLUT3 in neurons and GLUT1 in astrocytes, with different kinetic properties [17]. Astrocytes were seen to increase glucose transport and utilization in response to glutamergic activation. Likewise, neurons and astrocytes also express different LDH isoforms - astrocytes predominantly express LDH5, which produces lactate, while neurons express mostly LDH1, which essentially converts lactate to pyruvate, supporting the ANLSH model. Furthermore, lactate was shown to help maintain neuronal activity during periods of hypoglycemia and hypoxia [17].

There are experimental and computational data *for *as well as *against *the ANLSH - for example, some studies imply that neurons with basal activation show no net import of pyruvate or lactate [18], while Mangia and colleagues claim just the opposite of ANLSH, that is, neurons shuttle the lactate into astrocytes, and the only way this would work in reverse (ie astrocyte-to-neuron) is when the astrocytic glucose transport capacity is increased 12-fold [19]. As a matter of fact, it was shown that glutamate can stimulate glycolysis in astrocytes, by stimulating GLUT1 activity [20]. In this study, we model the brain energy metabolism of neurons and astrocytes using a computational model, incorporating genetic regulation of key transporters and enzymes. Since some key components (HK, GAPDH, PFK, PK, LDH, GLUT, MCT) of the metabolic network are regulated in an oxygen-dependent manner [[3,21]; and our data, see Results and Discussion], we have incorporated the hypoxia-dependent regulation of genetic networks to both neurons and astrocytes in our model. As a matter of fact, oxygen and glucose were shown to both act as signals for genetic regulation of certain regulatory molecules or enzymes in metabolic pathways - studies in liver, for instance, have shown that the glucose response element present within the pyruvate kinase (PK) promoter acts as a convergence point for HIF-1α, mediating crosstalk between glucose and oxygen signals [22]. It is successfully shown that hypoxia can in fact upregulate glucose transporters up to 12-fold in the astrocyte, as predicted by Mangia et al [19], supporting that ANLSH is feasible under energy-demanding conditions such as hypoxia. Under conditions of brain ischemia neurons were found to be more susceptible to damage than astrocytes, mainly because astrocytes tend to maintain large reserves of glycogen and can maintain glycolytic ATP synthesis for a considerably longer time than neurons [23]. Astrocytes were also shown to convert this glycogen into lactate, which is then transferred to neurons under periods of increased energy requirement or low glucose availability [23]. Furthermore, ischemic conditions of myocardial were shown to yield less ATP production and accumulation of intracellular lactate [24].

In this study, we have modeled (reactions and numerical values of the parameters are given in Additional File 1) both views separately and assessed their ATP production potential from a genetic regulation perspective, focusing only on the production of ATP and not consumption. It has to be emphasized that our model does not include any ATP sinks that mimic use of ATP in the cells, leading to non-physiological levels of ATP building up of the cell: we have purposefully done so, in order to clearly observe the accumulation of ATP over a period of time, since we are only comparing the conventional view vs lactate shuttle in terms of ATP production efficiency. Normally, neuronal cells use the ATP in a number of processes including electrical activity, transcription and translation, enzymatic events, motor proteins in the cell etc, but none of these events are included in this study so as to observe the effects of the shuttle on ATP production. It must be noted that hypoxia will also affect the metabolic rate of any cell, therefore ATP will be used to different extents, which would have complicated the interpretation of the results if incorporated to the model.

**Figure 1:**
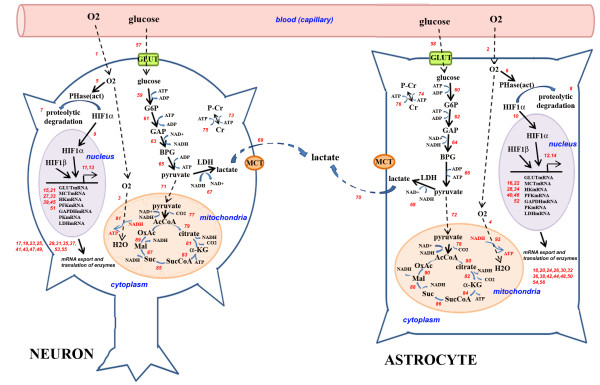
**The classical view of energy metabolism within neurons and astrocytes**. Blood glucose is transported and utilized **by both cells **in glycolysis, and the resulting pyruvate is mainly converted to AcetylCoA within the mitochondria, to be broken down in citric acid cycle, where NADH produced is converted to ATP in chemiosmosis. Some of the pyruvate is converted to lactate, however, and released into the extracellular matrix. Glucose or lactate transporters as well as certain glycolytic and other enzymes were modeled to be regulated by hypoxia. Transport across compartments are shown with dashed arrows. (Please note that the figures are simplified due to space constraints and not all reactions are explicitly included; please refer to Additional File 1 for full set of reactions modeled; numbers in red correspond to the reaction numbers in this file). GLUT, Glucose transporter; MCT, lactate transporter; HK, hexose kinase; PFK, phosphofructo kinase; GAPDH, glyceraldehyde-P-dehydrogenase; PK, pyruvate kinase; LDH, lactate dehydrogenase; AcCoA, Acetyl coenzyme A; a-KG, alpha-ketoglutarate; SucCoA, succinyl coenzyme A; Suc, succinate; Mal, malate; OxAc, oxaloacetate; ATP, adenosine triphosphate; NADH, G6P, glucose-6-phosphate; GAP, glyceraldehyde-3-phosphate; BPG, bisphosphoglycerate; PHase, pyrolyl hydroxylase; HIF, hypoxia-inducible factor; Cr, creatine; P-Cr, phospho-creatine.

In the first model, the classical view assumes that both neurons and astrocytes can take up glucose and use it in glycolysis and aerobic respiration (Figure 1). The pyruvate can choose two routes - some of it will be transported into mitochondria, converted into Acetyl Coenzyme A and enter the citric acid cycle, whereas some will be converted into lactate by lactate dehydrogenase (LDH) enzyme and secreted into the extracellular matrix through a generic monocarboxylate transporter, MCT (Figure 1).

**Figure 2:**
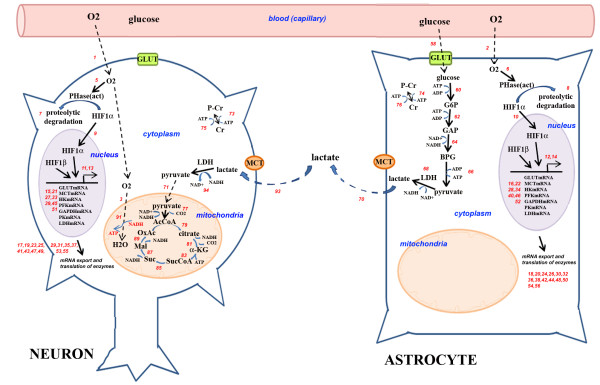
**The astrocyte-neuron lactate shuttle hypothesis (ANLSH)**. In this model, glucose is mainly utilized **by the astrocyte **in glycolysis, and the resulting pyruvate is converted to lactate and released to the extracellular matrix. This lactate is then taken up by the neuron, converted into pyruvate, and utilized in aerobic respiration within the mitochondria. Transport across compartments are shown with dashed arrows. (Please note that the figures are simplified due to space constraints and not all reactions are explicitly included; please refer to Additional File 1 for full set of reactions modeled; numbers in red correspond to the reaction numbers in this file). GLUT, Glucose transporter; MCT, lactate transporter; HK, hexose kinase; PFK, phosphofructo kinase; GAPDH, glyceraldehyde-P-dehydrogenase; PK, pyruvate kinase; LDH, lactate dehydrogenase; AcCoA, Acetyl coenzyme A; a-KG, alpha-ketoglutarate; SucCoA, succinyl coenzyme A; Suc, succinate; Mal, malate; OxAc, oxaloacetate; ATP, adenosine triphosphate; NADH, G6P, glucose-6-phosphate; GAP, glyceraldehyde-3-phosphate; BPG, bisphosphoglycerate; PHase, pyrolyl hydroxylase; HIF, hypoxia-inducible factor; Cr, creatine; P-Cr, phospho-creatine.

The second model, ANLSH, assumes that glucose is mainly taken up by the astrocyte and used up in glycolysis, the resulting pyruvate is converted into lactate by the astrocyte-specific LDH, and secreted out to the extracellular matrix via astrocyte-specific MCT. This lactate in turn is taken up by the neuron via the neuron-specific MCT, and converted into pyruvate via neuron-specific LDH, which is then free to enter the citric acid cycle in mitochondria (Figure 2).

In both models, some of the key enzymes or transporters were modeled to be regulated in an oxygen-dependent manner through Hypoxia Inducible Factor (HIF) both in neurons and astrocytes (Figure 1). Available oxygen levels are quite important for the survival of cells, and as such cells have devised methods to sense oxygen levels and respond accordingly. Heme-containing prolyl hydroxylase enzymes (PHase) sense the levels of oxygen, and under normoxic conditions interact with HIF1-α and hydroxylate it on Proline residues, labeling it for proteasome-dependent degradation [25]. Under hypoxic conditions, PHase cannot interact with HIF1-α, which then accummulates and translocates to the nucleus, where it regulates many hypoxia-inducible genes [25].

In this study we have investigated the effects of hypoxia-inducible transporters and enzymes, including GLUT, MCT, HK, GAPDH, PFK, PK and LDH (see Materials and Methods for details of the model), in the overall energetic output of either model. It should be emphasized again that this work focuses on the energetic output of the classical view vs ANLSH in the presence of hypoxia-dependent regulation of key enzymes, irrespective of glutamergic activation or stimulation. Our results show that the ANLSH is more advantageous for the neuron in terms of ATP produced, both under hypoxic and normoxic conditions, although it does not provide a significant advantage for the astrocyte. We therefore believe that rather than a "classical-OR-ANLSH" choice for the cells, neurons and astrocytes can switch between one model or the other, depending on the energy requirements of the neuron.

## Results and Discussion

### Hypoxia-dependent regulation of key metabolic enzymes

**Figure 3:**
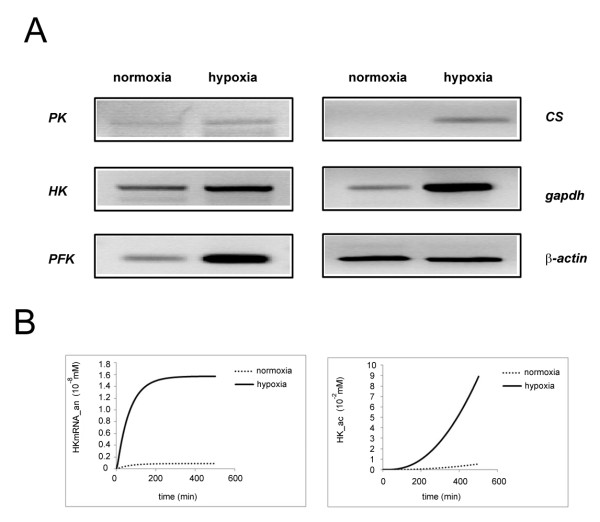
**Hypoxia-dependent regulation of key metabolic enzymes**. **(a) **Reverse transcription - Polymerase Chain Reaction (PCR) results from PC12 cells grown either in hypoxic or normoxic conditions. The transcript levels are studied for enzymes pyruvate kinase (PK), hexokinase (HK), phosphofructokinase (PFK), citrate synthase (CS), Glyceraldehyde dehydrogenase (GAPDH), and β-actin (as internal control). **(b) **The kinetics of HK mRNA and protein levels according to the classical model under normoxic and hypoxic conditions (normoxia = 7 mM O_2_, hypoxia = 0.35 mM O_2_) as an example of hypoxia-responsive gene expression in the model. HK mRNA production kinetics in astrocytes (^An^) under normoxic and hypoxic condition is shown on the left panel, and HK protein production kinetics in astrocytes (^Ac^) under normoxic and hypoxic condition is shown on the right panel. (Please note that all hypoxia-responsive genes have same rate equations, thus the same kinetic profiles in the model).

Hypoxia-responsive nature of some metabolic enzymes or transporters have been studied in different cell types such as liver cells, adipocytes, HeLas or COS cells, as discussed in Background, however the behavior of many of these enzymes are still not completely known in cells of the nervous system. In order to understand how some of the key enzymes behave under hypoxic conditions in neuron-like cells, we have used the PC12 cells, which are commonly used as neuronal differentiation model as they can undergo neuron-like physiological and molecular changes in response to Nerve Growth Factor (NGF) or other stimulants. To that end, we have studied enzymes such as Pyruvate Kinase (PK), Hexokinase (HK), and the rate-limiting enzyme phosphofructokinase (PFK), as well as the first enzyme of the aerobic respiration, citrate synthase (CS), that converts oxaloacetic acid and acetyl coA to citric acid in the cycle. Intriguingly, all of these enzymes have shown hypoxia-induced upregulation in transcription, albeit to different extents (Figure 3a). Glyceraldehyde dehydrogenase (GAPDH) enzyme is generally used as an internal control in RT-PCR reactions, however since it is itself one of the enzymes of glycolysis, we have used two different internal controls, GAPDH and β-actin, a cytoskeletal component. We have found, to great surprise, that widely-accepted internal control standard, GAPDH itself, was hypoxia-induced, when the cDNAs were normalized with respect to β-actin control (Figure 3a).

For that reason, as well as other reports in the literature discussed above, we had incorporated such hypoxia-dependent regulation to the transcription module of many metabolic enzymes (see Materials and Methods for details), and studied ATP production under normoxic vs hypoxic conditions for the first time in this study. We have next confirmed that our model indeed gives us hypoxia-induced upregulation of these enzymes at both mRNA and protein synthesis levels; the transcript and protein of these enzymes were confirmed to respond to hypoxia as expected (Figure 3b shows HK as an example; it should be noted that all hypoxia-responsive genes listed in Additional File 1 show the same kinetic profile upon simulation). Since at this point we do not have absolute kinetic parameters for the hypoxic regulation of each promoter separately, in the model we have assumed similar hypoxia-response kinetics, as shown in detail in Additional File 1 and explained in Materials and Methods.

### The energy efficiency of the classical view under both normoxic and hypoxic conditions

**Figure 4:**
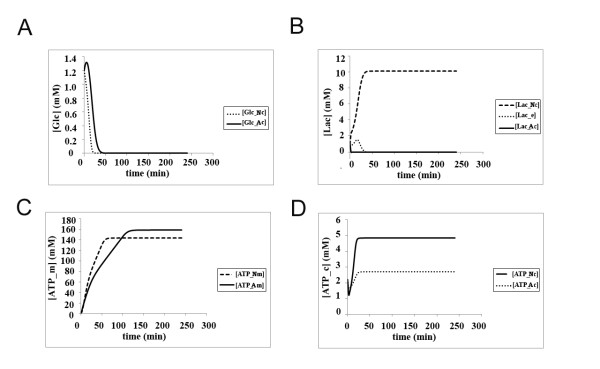
**The kinetics of glucose utilization, lactate production and ATP synthesis in neurons and astrocytes according to the classical model under normoxic condition (Initial glucose concentration in the blood = 4.56 mM, oxygen concentration = 7 mM) **(a) **Glucose consumption in neurons (^Nc^) and astrocytes (^Ac^); **(b) **Lactate kinetics in neurons (^Nc^), astrocytes (^Ac^) and the extracellular matrix (^e^); **(c) **Mitochondrial ATP production in neurons (^Nm^) and astrocytes (^Am^); **(d) **Cytoplasmic ATP production in neurons (^Nc^) and astrocytes (^Ac^)**.

We first investigated the energy efficiency of the classical model under two different oxygen concentrations. Under normoxic conditions in the classical model, glucose is readily consumed, within the first 50 min, in both astrocytes and neurons (Figure 4a). This pathway results in the production of lactate in both cell types, but although lactate is quickly discarded out of the astrocyte within around 30 min, very little lactate can be transported out of the neuron due to the high amount of lactate buildup in neuronal cytoplasm (Figure 4b). Very little extracellular lactate accumulates under these conditions (Figure 4b). It should be noted that, in our model, extracellular lactate is not shuttled into the vascular endothelial cells of the capillary, so as to focus on lactate shuttle between only two cell types, the neuron and the astrocyte. Under normoxia, most of the ATP is produced within the mitochondria around the same level in both neurons (reaching a plateau of around 160 mM) and astrocytes (reaching a plateau of around 140 mM) (Figure 4c). The levels of ATP produced in the cytoplasm of astrocytes and neurons, however, are different - neurons can produce up to 5 mM of ATP in cytoplasm through glycolysis, whereas astrocytes can merely generate around 2.5 mM of ATP in their cytoplasm (Figure 4d). This is not only a reflection of different volumes of cytoplasm in astrocytes and neurons, since mitochondrial volumes differ by a similar ratio between the two cell types and yet the amount of mitochondrial ATP synthesis is not significantly different (see Additional File 1 for compartmental volumes).

**Figure 5:**
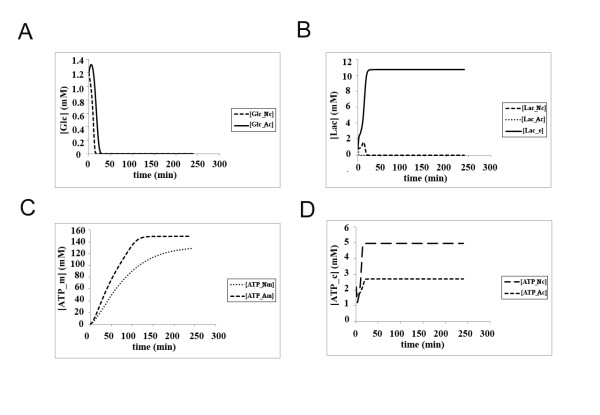
**The kinetics of glucose utilization, lactate production and ATP synthesis in neurons and astrocytes according to the classical model under hypoxic condition (Initial glucose concentration in the blood = 4.56 mM, oxygen concentration = 0.35 mM); **(a) **Glucose consumption in neurons (^Nc^) and astrocytes (^Ac^); **(b) **Lactate kinetics in neurons (^Nc^), astrocytes (^Ac^) and the extracellular matrix (^e^); **(c) **Mitochondrial ATP production in neurons (^Nm^) and astrocytes (^Am^); **(d) **cytoplasmic ATP production in neurons (^Nc^) and astrocytes (^Ac^)**.

When both cells are exposed to hypoxic conditions, glucose consumption is not changed significantly (Figure 5a) in spite of the fact that GLUT and other enzymes are overexpressed in an oxygen-dependent manner in the model (Figure 3b), whereas lactate kinetics changes radically - very little lactate accumulates in both neuronal and astrocytic cytoplasm, but the amount of extracellular lactate rises to around 10 mM (Figure 5b). Although the conditions are hypoxic, the mitochondria can still carry out citric acid cycle to a large extent, but still the mitochondrial ATP production in the neuron is slightly reduced to 120 mM in the neuron and 140 mM in the astrocyte at steady-state conditions (Figure 5c). The cytoplasmic ATP levels are unaffected by the oxygen levels, as expected from anaerobic glycolysis (Figure 5d).

### The energy efficiency of the astrocyte-neuron lactate shuttle hypothesis under normoxic, hypoxic, and glucose starvation conditions

Next, the ANLSH is modeled as described in Figure 2, where glucose is essentially taken up by the astrocyte and consumed in glycolysis until pyruvate, which is then converted into lactate and transported into the extracellular matrix (Figure 2). Extracellular lactate is then taken up by the neuron, converted to pyruvate and entered into aerobic respiration in the neuron (Figure 2, see Materials and Methods for details).

**Figure 6:**
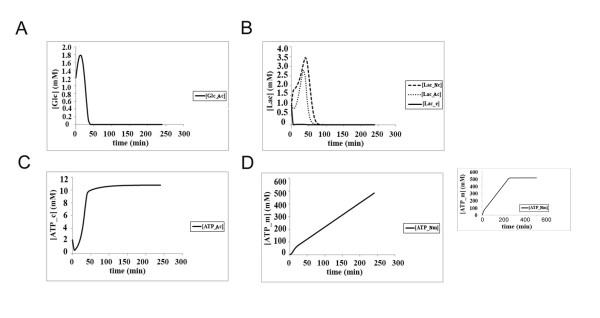
**The kinetics of glucose utilization, lactate production and ATP synthesis in neurons and astrocytes according to the ANLSH under normoxic condition (Initial glucose concentration in the blood = 4.56 mM, oxygen concentration = 7 mM); **(a) **Glucose consumption in astrocytes (^Ac^); **(b) **Lactate kinetics in neurons (^Nc^), astrocytes (^Ac^) and the extracellular matrix (^e^); **(c) **ATP production in astrocyte cytoplasm (^Ac^); **(d) **Mitochondrial ATP production in neurons (^Nm^); inset shows results from a longer simulation (500 min)**.

Under normoxic conditions with normal levels of glucose, glucose intake is slightly increased in astrocytes (Figure 6a) as compared to that in classical model (Figure 4a). On the other hand, since lactate generated by the astrocytes are transferred to the neurons to be converted into pyruvate, there is no build-up of lactate in the neuronal cytoplasm, unlike the steady-state levels of around 10 mM cytoplasmic lactate in the classical view (Figure 4b), and the relatively low levels of cytoplasmic lactate in neurons rapidly declines within 1 hr to tolerable levels with the ANLSH model (Figure 6b). Under these conditions, the cytoplasmic ATP production in the astrocyte (just over 10 mM at steady-state; Figure 6c) is higher than that in the classical view (around 2.5 mM at steady-state; Figure 6d). The mitochondrial ATP production within neurons, however, is enhanced by over 3-fold in the ANLSH (over 500 mM as opposed to around 150 mM; compare Figure 6d and 4c, respectively). It should be noted that neuronal mitochondrial ATP levels reach the steady-state at 500 mM abruptly at around 300 min (inset to Figure 6d).

**Figure 7:**
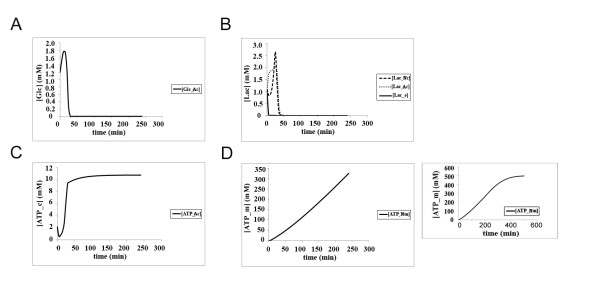
**The kinetics of glucose utilization, lactate production and ATP synthesis in neurons and astrocytes according to the ANLSH under hypoxic condition (Initial glucose concentration in the blood = 4.56 mM, oxygen concentration = 0.35 mM); **(a) **Glucose consumption in astrocytes (^Ac^); **(b) **Lactate kinetics in neurons (^Nc^), astrocytes (^Ac^) and the extracellular matrix (^e^); **(c) **ATP production in astrocyte cytoplasm (^Ac^); **(d) **Mitochondrial ATP production in neurons (^Nm^); inset shows a longer simulation (500 min)**.

When the cells are simulated under hypoxic conditions with the ANLSH model, it is observed that while glucose consumption in the astrocyte does not seem to be affected greatly (Figure 7), the amount of lactate produced declines slightly (2 mM in the astrocyte as opposed to 2.5 mM in normoxic conditions; Figure 7b vs Figure 6b, respectively). The ATP produced in the astrocyte cytoplasm does not change, reaching the same steady-state of 10 mM within 50 min (Figure 7c). The amount of ATP synthesized in the neuronal mitochondria has a slower rate, reaching 300 mM by 250 min (Figure 7d), however when simulation is run for longer periods it is observed that even under hypoxic conditions the steady-state levels of 500 mM are reached but only at around 500 min (inset to Figure 7d).

**Figure 8:**
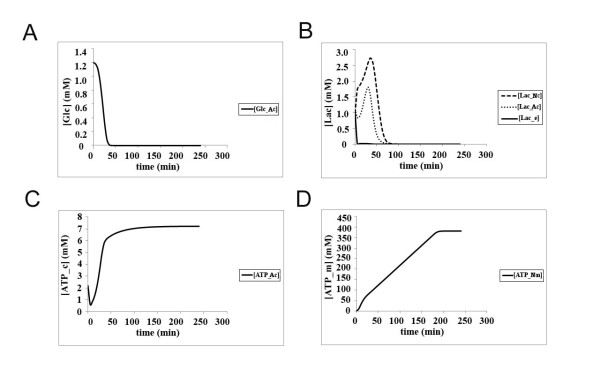
**The kinetics of glucose utilization, lactate production and ATP synthesis in neurons and astrocytes according to the ANLSH for starvation conditions (Initial glucose concentration in the blood = 1 mM (glucose starvation), oxygen concentration = 7 mM); **(a) **Glucose consumption in astrocytes (^Ac^); **(b) **Lactate kinetics in neurons (^Nc^), astrocytes (^Ac^) and the extracellular matrix (^e^); **(c) **ATP production in astrocyte cytoplasm (^Ac^); **(d) **Mitochondrial ATP production in neurons (^Nm^)**.

When the cells are placed in normoxic conditions with glucose starvation, ie constant flow of 1 mM blood glucose, glucose transport into the astrocyte is largely compromised due to higher intracellular glucose levels, which is rapidly consumed (Figure 8a), leading to also a lower level of lactate (Figure 8b) and ATP (Figure 8c and Figure 8d) production in the astrocyte as compared with Figure 6c and 7c). However, when the amount of lactate taken up by the neuron is analyzed, the lactate levels are seen to peak at around 2.5 mM under both normoxic and hypoxic conditions irrespective of blood glucose levels (Figure 7b and Figure 8b), albeit slightly lower than that in normoxic conditions (around 3.5 mM, Figure 6b). Nevertheless, the mitochondrial ATP production in the neuron shows a much different profile under hypoxic vs normoxic-starvation conditions: whereas under hypoxic conditions ATP levels reach 500 mM at around 500 min, albeit with lower rate (inset to Figure 6d and Figure 7d, respectively), under normoxic-starvation conditions the ATP production shows a biphasic profile, with a rapid increase in the first 20 min, then accumulating with a slower rate up until 200 min, at which point it reaches a steady-state at 400 mM (Figure 8d).

## Discussion

Our results indicate that under all three conditions studied (normal glucose and normoxia; normal glucose and hypoxia; low glucose and normoxia), ANLSH model provides the neuron with on average around 3-fold more mitochondrial ATP than under normoxia. Cytoplasmic ATP production in the astrocyte is also much more using the ANLSH, around 2- to 4-fold, however it should be noted that in ANLSH it is assumed that there is no mitochondrial ATP production, hence the overall astrocytic ATP production is significantly reduced (around 150 mM using classical model vs around 10 mM using ANLSH). Oxygen and glucose deprivation (OGD) was previously shown to decrease neuronal NADH levels but not astrocytic ones, and neurons were seen to be more susceptible to OGD-mediated cell death [26]. In the same study, it was shown that hypoxia was not detrimental to cells, but lack of glucose was more crucial - indeed in our simulations normoxia vs hypoxia does not change the levels of ATP significantly, whereas decrease in glucose concentration has a serious negative effect.

It must be emphasized that in this model glucose is the limiting reactant, in other words it is not fed into the blood continuously; furthermore the model is a time course simulation not steady state, and there is no feedback inhibition on the glycolytic pathway. Therefore at the end of the simulations glucose concentration decreases as ATP gets produced. On the other hand, lactate accumulates in the extracellular matrix, therefore intracellular concentration decreases, or it shuttles into the neuron and gets converted to pyruvate hence its intracellular concentration decreases

Astrocytes were indeed reported to have 1 or 2 mitochondria [27], neurons have 10s of mitochondria [28], which significantly increase the amount of ATP produced in the neuron. In the present study all mitochondrial activity was considered to be concentrated in a single sub-compartment representing one mitochondrion per cell (be it neuron or astrocyte). It should be also noted that in the recent views of the shuttle hypothesis, astrocyte mitochondria are not considered to be completely inactive; however the kinetic parameters regarding this situation are not yet absolutely known at the single cell level, therefore we have considered complete shutdown of mitochondria in astrocytes. Under these conditions, the amount of ATP produced in the astrocyte with the ANLSH under any condition is very low, this ATP can not sustain normal astrocytic functions for very long, however it is certain that a temporary ANLSH would benefit the neuron enormously even with a single mitochondrion; the output will be much higher for a neuron with multiple mitochondria seen *in vivo*. Therefore, we would like to propose that there is no strict classical-or-ANLSH model choice in the brain, but rather a switch based on energy demand of the neuron. It is also equally likely that unlike in this model astrocytes do not completely switch off their aerobic respiration, but rather change the ratio of pyruvate that is converted to lactate, thus using an intermediate system between the classical view and the ANLSH.

## Conclusions

In this study, we have demonstrated that the ANLSH is more advantageous for the neuron in terms of ATP produced, both under hypoxic and normoxic conditions, although it does not provide a significant advantage for the astrocyte. We therefore believe that rather than a "classical-OR-ANLSH" choice for the cells, neurons and astrocytes can switch between one model or the other, depending on the energy requirements of the neuron. However, more detailed, genome-wide kinetic models will surely prove useful in analyzing these models in more detail as well as understanding such an energy demand-dependent switching [29].

## Methods

### COPASI modeling platform

COPASI 4.4.29 (COmplex PAthway SImulator) software package was used for analysis [30]. In deterministic modeling, the program solves differential equations using the routine LSODE (Livermore Solver of Ordinary Differential Equation).

### Multi-Compartmental Models

To simulate the metabolic processes that occur inside neuron and astrocyte during normoxia and hypoxia, a general mathematical model was developed where cells have interaction between capillary and extracellular area with distinct volume of nucleus, cytosol and mitochondrion domains (Figures 1 and 2). For the sake of simplicity, total activity of the mitochondria were described as a single sub-compartment both in neuron and astrocyte. Compartment volumes are given in explanation of Additional File 1. The compartment volumes are the same in both models: *V^Nn ^*= 0.033 L, V^Nc ^= 0.33 L, V^Nm ^= 0.0855 L, *V^An ^*= 0.019 L, *V^Ac ^*= 0.19L, V^Am ^= 0.0475 L, *V^e ^*= *0.2 *L, *V^c ^*= *0.095 *L. ANLSH hypothesis suggests no itochondrion in the astrocytes, therefore the astrocyte mitochondrion volume is pertinent to the classical model only [31-34]. Reactions with number 1-92 are pertinent to model 1. Reactions with number 1-14, 16, 18-26, 28, 30, 32, 34, 36, 38, 40, 42, 44, 46, 48, 50-56, 58, 60, 62, 64, 66, 68, 70-71, 73, 75, 77, 79, 81, 83, 85, 87, 89, 91, 93-94 are pertinent to model 2 (Additional File 1). Between compartments (capillary-cytosol, cytosol-mitochondrion, and nucleus-cytosol) molecular transport was assumed to occur either by passive diffusion or carrier-mediated transport between domains x and y and the transport rate equations are given in Equations 1 and 2, respectively. And all other reactions (such as X+Y → Z+W) in cells were assumed to obey Michaelis-Menten kinetics rate law (Eqn.3) [20,21].

### Transport Phenomena between compartments

#### a) Passive diffusion (O_2_, CO_2_)

Passive diffusion is linearly related to substrate concentrations on both sides of the cell membrane. However, since this diffusion is a nonsaturable process, membrane transport coefficient, membrane permeability and effective surface area are important parameters in transport process and seen in the equation.

(1) $$J_{x\to y}=\gamma_{x\to y,j}\left(C_{x,j}-\sigma_{x\to y,j}C_{y,j}\right)$$

where *γ*_*x*→*y, j *_is the membrane transport coefficient and *σ*_*x*→*y, j *_is the partition coefficient. Cx, j and Cy, j are compartmental concentrations of species j.

#### b) Facilitated transport (glucose, lactate, pyruvate)

The rate of the facilitated transport can be defined by using Michaelis Menten enzyme kinetics where V_x→y, j _is the transport rate coefficient, K_m, x→y, j _is the affinity coefficient and C_x, j _is concentration of j at x compartment.

(2) $$J_{x\to y,j}=\frac{V_{x\to y,j}C_{x,j}}{K_{m,x\to y,j}+C_{x,j}}$$

### Kinetics of Individual reaction steps

(3) $$J=J_{max,x}\left(\frac{C_{x}C_{y}}{K_{x-y,z-w}+C_{x}C_{y}}\right)$$

Numerical values of the biochemical parameters were obtained mainly from previous experimental reports (Additional File 1) and initial concentrations of the metabolites (Additional File 1) were obtained from literature. Where no experimental data were available, mathematical estimates, either from computational reports or from our own estimations, were used in the models. The detailed biochemical reactions for the two models (classical view and ANLSH) in each cell are defined and initial metabolite concentrations used for the two models are listed in Additional File 1.

In this study, the energy metabolism in neuron and astrocyte is investigated from two different perspectives. One model is from the point of classical view (1st Model, Additional File 1) and the other is from the point of Astrocyte-Neuron lactate shuttle hypothesis (ANLSH, 2nd Model, Additional File 1). For both models, we have analyzed the time-course data and results were imported to MS Excel, and graphs have been generated using MS Excel.

### The Model

The details of both models are given in Additional File 1 and the framework is given in Figures 1 and 2. The metabolic part of the model is essentially based on the model of Aubert and Costalat and Zhou et al., with the exception of ion channels and neuronal stimulation [33,34]. The hypoxia-dependent genetic regulation aspects are modeled based on the work of Yucel and Kurnaz [31].

In short, the classical view states that both neurons and astrocytes can take up glucose from the blood through a generic glucose transporter, GLUT, and use it in glycolysis. Glucose is activated by addition of two phosphates from ATP hydrolysis through action of Hexokinase (HK) and phosphofructokinase (PFK), and broken down (or "lysed") to two glyceraldehyde-3-phosphates (GAP), to be ultimately converted into pyruvate, generating 2 ATPs and 1 NADH from each GAP (Figure 1). The NADH is generated by the action of GAP dehydrogenase, or GAPDH, and one of the ATPs is produced at the last step by pyruvate kinase, or PK. The pyruvate then enters two different routes - some of it will be transported into mitochondria, converted into Acetyl Coenzyme A and enter the citric acid cycle, whereas some will be converted into lactate by a generic lactate dehydrogenase (LDH) enzyme and secreted into the extracellular matrix through a generic monocarboxylate transporter, MCT (Figure 1). In either cell, some of the above-mentioned key enzymes or transporters, ie GLUT, PFK, GAPDH, PK, LDH and MCT [22,18] are regulated in an oxygen-dependent manner through HIF transcription factor (Figure 1).

In the astrocyte-neuron lactate shuttle hypothesis (ANLSH), glucose is mainly taken up by the astrocyte through the astrocyte-specific GLUT and used up in glycolysis, the resulting pyruvate is converted into lactate by the astrocyte-specific LDH, and secreted out to the extracellular matrix via astrocyte-specific MCT. This lactate in turn is taken up by the neuron via the neuron-specific MCT, and converted into pyruvate via neuron-specific LDH, which is then free to enter the citric acid cycle in mitochondria (Figure 2). This model, too, incorporates oxygen-dependent regulation of some of the enzymes and transporters as discussed in the first model above.

In both models, the mitochondrial reactions are modeled in a similar manner; namely, pyruvate is taken into the mitochondria, converted into Acetyl coenzyme A, and entered into the citric acid cycle. The cycle produces GTP (assumed in this model to be essentially equivalent to ATP), NADH and FADH2 (Figure 1). The NADH and FADH2 is used as electron donors in the electron transport chain (ETC), to ultimately produce ATP (Figure 1); a simplified equation based on previous models was used for modeling ETC (Additional File 1) [6,34,35].

### Experimental study of hypoxia-dependent gene regulation

PC12 cells were maintained in DMEM containin 10% Horse serum, 5%FBS, 1X L-Glutamine and 1X Penicillin/Streptomycin. For hypoxia studies, cells were transferred to hypoxia incubator corresponding to 2% O_2_. RNA was isolated from cells 3 days after plating (plating density: 10^5^cells/ml), following manufacturer's instructions (Roche, High Pure RNA Isolation Kit). cDNA was synthesized using Promega, ImProm-II™Reverse Transcription System Kit. PCR reaction was carried out using the primers listed in Table 1, at the indicated conditions (typically, the reaction was performed in 30 ul, with 0.5 ul dNTPs and 1 ul of each primer):

**Table 1:** List of primers used in RT-PCR reactions.

Transcript amplified	Primer sequence (F 5'to 3'; R 5'to 3')	Product length	Tm (°C)	Cycle #
PK (pyruvate kinase)	F: AGTCGGAGGTGGAAATTGTG R. AGGTCCACCTCAGTGTTTGG	267 bp	60	45
HK (hexokinase)	F: CAGGGTCTGAGCAAGGAGAC R: GCTTCCTTCAGCAAGGTGAC	430 bp	60	45
PFK(phosphofructokinase)	F: CACCATCAGCAACAATGTCC R: AGTCGTGGATGTTGAAAGGG	242 bp	60	40
GAPDH (Glyceraldehydephosphate dehydrogenase)	F: TCG GAG TCA ACG GAT TTG G R: GCA TTG CTG ATG ATC TTG AGG	500 bp	50	30
CS (citrate synthase)	F: AAGGCTAAAGGTGGGGAAGA R: CCATTCATAGCTGCTGCAAA	565 bp	54	35
Beta-actin	F: GGCTTTAGGAGCTTGACAATACTG R: GCATTGGTCACCTTTAGATGGA	511 bp	60	30

## Abbreviations

x, y: compartments; C_x, j _: concentration of j in x; J_x→y, j _: transport rate; γ_x→y, j _: membrane transport coefficient; *σ*_*x*→*y, j *j _: partition coefficient; V_x→y, j _: transport rate coefficient; K_x→y, j _: affinity coefficient; Glc: glucose; GLUT: glucose transporter; MCT: lactate transporter; HK: hexokinase; PFK: phosphofructokinase; GAPDH: glyceraldehyde-P-dehydrogenase; PK: pyruvate kinase; LDH: lactate dehydrogenase; AcoA: Acetyl coenzyme A; a-KG: alpha-ketoglutarate; SucCoA: succinyl coenzyme A; Suc: succinate; Mal: malate; OxAc: oxaloacetate; ATP: adenosine triphosphate; NADH: nicotinamide adenine dinucleotide; FADH2: flavin adenine dinucleotide; GTP: guanosine-5'-triphosphate; ETC: electron transport chain; G6P: glucose-6-phosphate; GAP: glyceraldehyde-3-phosphate; BPG: bisphosphoglycerate; PHase: pyrolyl hydroxylase; HIF: hypoxia-inducible factor; Cr: creatine; P-Cr: phospho-creatine; PD: Passive/facilitated diffusion; MM: Michaelis Menten; HMM: Henri Michaelis Menten; UI: Uncompetitive inhibition; MA: Mass action; ^N ^: neuron; ^A ^: astrocyte; ^c ^: cytosol; ^n ^: nucleus; ^m ^: mitochondrion; ^b ^:blood (used interchangibly with "capillary"); ^e ^:extracellular area

## Authors' contributions

SG: has performed the simulations, generated the graphs and helped write the manuscript; IAK: has helped develop the model, interpreted the results and written the paper; MO: has helped develop the model, interpreted the results and helped write the manuscript. All of the authors have read and approved of the manuscript.

## Supplementary Material

Additional file 1 **Equations which appear in both Model 1 and 2. Biochemical reactions and kinetic parameters according to classical view (Model 1) and ANLSH (Model 2)**. This file contains all the reactions and equations used in the simulation of both models, as well as the kinetic parameters and the references thereof. Click here for file
