# Supplementary material for: Astrocyte - neuron lactate shuttle may boost more ATP supply to the neuron under hypoxic conditions - in silico study supported by in vitro expression data
Source: BMC Syst Biol. 2011 Oct 13;5:162. doi: 10.1186/1752-0509-5-162 (PMC3202240; doi:10.1186/1752-0509-5-162)
Supplement: Additional file 1 — Equations which appear in both Model 1 and 2. Biochemical reactions and kinetic parameters according to classical view (Model 1) and ANLSH (Model 2). This file contains all the reactions and equations used in the simulation of both models, as well as the kinetic parameters and the references thereof. [file 1752-0509-5-162-S1.DOC]

# Tables

**Table 1. Equations which appear in both Model 1 and 2 Biochemical reactions and kinetic parameters according to classical view (Model 1) and ANLSH view (Model 2).** The compartment volumes are the same in both models: $V^{\text{Nn}}=0.033$L, L, $V^{\text{Nm}}=0.0855$ L, $V^{\text{An}}=0.019$ L, $V^{\text{Ac}}=0.19$L, L, $V^{e}=0.2$ L, $V^{b}=0.095$ L. ANLSH hypothesis suggests no mitochondrion in the astrocytes, therefore the astrocyte mitochondrion volume is pertinent to the classical model only. Reactions with number 1-92 are pertinent to model 1. Reactions with number 1-14,16,18-26,28,30,32,34,36,38,40,42,44,46,48,50-56,58,60,62,64,66,68,70-71,73,75,77,79,81,83,85,87,89,91,93-94 are pertinent to model 2. (^b^:blood; ^N^:neuron; ^A^: astrocyte; ^e^:extracellular area; ^n^:nucleus; ^m^:mitochondria; V^nn^: volume of neuron nucleus, V^an^: volume of astrocyte nucleus; UI: uncompetitive inhibition (J=V*S/(Km+S*(1+Inhibitor/Ki))); PD: passive diffusion, defined in Eqn.1, Materials and Methods; MM: Michaelis-Menten ()**; HMM: Henri-Michaelis-Menten ()**; NTP: nucleoside triphosphate, or nucleotides; Phase: prolyl hydroxylase; act: active; HIFa: HIF-1α; HIFb: HIF-1β; HIFab: HIF-1α / HIF-1β complex; aa: amino acid; RNApoly: RNA polymerase; *** equations are examples to pre-defined kinetic types in Copasi and although conceptually synonymous, they refer to different equation constructions; all other kinetic types not explicitly defined in the Table or text are built-in kinetic types of COPASI*)

| # | Reaction | Reaction type | Kinetic parameters | Initial Concentration (mM) | Modifier | References |
| --- | --- | --- | --- | --- | --- | --- |
| 1 |  | *PD* | *A=0.1463 l/min, a= 1* |  |  | [33] |
| 2 |  | *PD* | *A=0.1463 l/min, a= 1* |  |  | *[33]* |
| 3 |  | *PD* | *A=0.5 l/min, a= 1* |  |  | *[33]* |
| 4 |  | *PD* | *A=0.2926l/min, a= 1* |  |  | *[33]* |
| 5 |  | *MM* | *Kcat=3.4617min^-1^,Km=0.24mM* |  | *O_2_* | *[30]* |
| 6 |  | *MM* | *Kcat=1.9931min^-1^, Km=0.24mM* |  | O_2_ | [30] |
| 7 |  | *MM* | *Kcat=2.51e014min^-1^, Km=0.002mM* |  | PHase_act_ | [30], EC3.1.2.15 (ExPaSy) |
| 8 |  | *MM* | *Kcat=1.44e014min^-1^, Km=0.002mM* |  | PHase_act_ | [30], EC3.1.2.15 (ExPaSy) |
| 9 |  | *Uİ* | *Km=0.004mM, V=0.56mMol/min,*  *Ki=1e-09mMol/l* |  | O_2_ | Estimation, [30] |
| 10 |  | *Uİ* | *Km=0.004mM,V=0.3249mMol/min,*  *Ki=1e-9mMol/l* |  | O_2_ | Estimation, [30] |
| 11 |  | *MA* | *K1=0.33, k2=9900* |  |  | [30] |
| 12 |  | *MA* | *K1=0.19, k2=5700* |  |  | [30] |
| 13 |  | *MA* | *k1=0.33, k2=6.6* |  |  | Estimation, [30] |
| 14 |  | *MA* | *k1=0.19, k2=3.8* |  |  | Estimation, [30] |
| 15 |  | *MM* | *Kcat=0.95min^-1^,Km=0.4mM* |  | RNApolyact | [30, EC2.7.7.6 (ExPaSy) |
| 16 |  | MM | *Kcat=0.547min^-1^,Km=0.4mM* |  | RNApolyact | [30], EC2.7.7.6 (ExPaSy) |
| 17 |  | HMM | *V=6e-05min^-1^,Km=0.004mM* |  |  | [30,31] |
| 18 |  | HMM | V=6e-05min^-1^,Km=0.004mM |  |  | [30,31] |
| 19 |  | MM | kcat=1603.8min^-1^,Km=0.022mM |  | GLUT3mRNA | [31,40], EC3.4.14.1 (ExPaSy) |
| 20 |  | MM | kcat=923.4min^-1^,Km=0.022mM |  | GLUT1mRNA | [30], EC3.4.14.1 (ExPaSy) |
| 21 |  | MM | *Kcat=0.95min^-1^,Km=0.4mM* |  | RNApolyact | [30], EC2.7.7.6 (ExPaSy) |
| 22 |  | MM | *Kcat=0.547min^-1^,Km=0.4mM* |  | RNApolyact | [30], EC2.7.7.6 (ExPaSy) |
| 23 |  | HMM | V=6e-05min^-1^,Km=0.004mM |  |  | [30,31] |
| 24 |  | HMM | V=6e-05min^-1^,Km=0.004mM |  |  | [30,31] |
| 25 |  | MM | kcat=1603.8min^-1^,Km=0.022mM |  | MCTmRNA | [30], EC3.4.14.1 (ExPaSy) |
| 26 |  | MM | kcat=923.4min^-1^,Km=0.022mM |  | MCTmRNA | [30], EC3.4.14.1 (ExPaSy) |
| 27 |  | MM | *Kcat=0.95min^-1^,Km=0.4mM* |  | RNApolyact | [30], EC2.7.7.6 (ExPaSy) |
| 28 |  | MM | *Kcat=0.547min^-1^,Km=0.4mM* |  | RNApolyact | [30], EC2.7.7.6 (ExPaSy) |
| 29 |  | HMM | V=6e-05min^-1^,Km=0.004mM |  |  | [30,31] |
| 30 |  | HMM | V=6e-05min^-1^,Km=0.004mM |  |  | [30,31] |
| 31 |  | MM | kcat=1603.8min^-1^,Km=0.022mM |  | HKmRNA | [30], EC3.4.14.1 (ExPaSy) |
| 32 |  | MM | kcat=923.4min^-1^,Km=0.022mM |  | HKmRNA | [30], EC3.4.14.1 (ExPaSy) |
| 33 |  | MM | *Kcat=0.95min^-1^,Km=0.4mM* |  | RNApolyact | [30], EC2.7.7.6 (ExPaSy) |
| 34 |  | MM | *Kcat=0.547min^-1^,Km=0.4mM* |  | RNApolyact | [30], EC2.7.7.6 (ExPaSy) |
| 35 |  | HMM | V=6e-05min^-1^,Km=0.004mM |  |  | [30,31] |
| 36 |  | HMM | V=6e-05min^-1^,Km=0.004mM |  |  | [30,31] |
| 37 |  | MM | kcat=1603.8min^-1^,Km=0.022mM |  | PFKmRNA | [30], EC3.4.14.1 (ExPaSy) |
| 38 |  | MM | kcat=923.4min^-1^,Km=0.022mM |  | PFKmRNA | [30], EC3.4.14.1 (ExPaSy) |
| 39 |  | MM | *Kcat=0.95min^-1^,Km=0.4mM* |  | RNApolyact | [30], EC2.7.7.6 (ExPaSy) |
| 40 |  | MM | *Kcat=0.547min^-1^,Km=0.4mM* |  | RNApolyact | [30], EC2.7.7.6 (ExPaSy) |
| 41 |  | HMM | V=6e-05min^-1^,Km=0.004mM |  |  | [30] |
| 42 |  | HMM | V=6e-05min^-1^,Km=0.004mM |  |  | [30,31] |
| 43 |  | MM | kcat=1603.8min^-1^,Km=0.022mM |  | GAPDHmRNA | [30], EC3.4.14.1 (ExPaSy) |
| 44 |  | MM | kcat=923.4min^-1^,Km=0.022mM |  | GAPDHmRNA | [30], EC3.4.14.1 (ExPaSy) |
| 45 |  | MM | *Kcat=0.95min^-1^,Km=0.4mM* |  | RNApolyact | [30], EC2.7.7.6 (ExPaSy) |
| 46 |  | MM | *Kcat=0.547min^-1^,Km=0.4mM* |  | RNApolyact | [30], EC2.7.7.6 (ExPaSy) |
| 47 |  | HMM | V=6e-05min^-1^,Km=0.004mM |  |  | [30,31] |
| 48 |  | HMM | V=6e-05min^-1^,Km=0.004mM |  |  | [30,31] |
| 49 |  | MM | kcat=1603.8min^-1^,Km=0.022mM |  | PKmRNA | [30], EC3.4.14.1 (ExPaSy) |
| 50 |  | MM | kcat=923.4min^-1^,Km=0.022mM |  | PKmRNA | [30], EC3.4.14.1 (ExPaSy) |
| 51 |  | MM | *Kcat=0.95min^-1^,Km=0.4mM* |  | RNApolyact | [30], EC2.7.7.6 (ExPaSy) |
| 52 |  | MM | *Kcat=0.547min^-1^,Km=0.4mM* |  | RNApolyact | [30], EC2.7.7.6 (ExPaSy) |
| 53 |  | HMM | V=6e-05min^-1^,Km=0.004mM |  |  | [30,31] |
| 54 |  | HMM | V=6e-05min^-1^,Km=0.004mM |  |  | [30,31] |
| 55 |  | MM | kcat=1603.8min^-1^,Km=0.022mM |  | LDHmRNA | [30], EC3.4.14.1 (ExPaSy) |
| 56 |  | MM | kcat=923.4min^-1^,Km=0.022mM |  | LDHmRNA | [30], EC3.4.14.1 (ExPaSy) |
| 57 |  | MM | V=0.45mM/min,kcat=4275,  Km=4MM |  | GLUT3_nc | [33] |
| 58 |  | MM | V=0.45mM/min,kcat=4275,  Km=4mM |  | GLUT1_ac | [33] |
| 59 |  | MM | V=1.21mM/min, kcat=39930,  Km=4mM | , | HK | [33] |
| 60 |  | MM | V=1.21mM/min kcat=22990,  Km=4mM | , | HK | [33] |
| 61 |  | MM | V=1.45mM/min,kcat=47850,  Km=0.86mM | , | PFK | [33] |
| 62 |  | MM | V=1.45mM/min, kcat=27550,  Km=0.86mM | , | PFK | [33] |
| 63 |  | MM | V=2.90mM/min, kcat=95700,  Km=0.13MM | , | GAPDH | [33] |
| 64 |  | MM | V=2.90mM/min, kcat=55100,  Km=0.13mM | , | GAPDH | [33] |
| 65 |  | MM | V=2.90mM/min, kcat=95700,  Km=0.2mM | , | PK | [33] |
| 66 |  | MM | V=2.90mM/min, kcat=55100,  Km=0.2mM | ,, | PK | [33] |
| 67 |  | MM | V=4.75x10^10^mM/min, kcat=100320,  Km=0.06mM | , | LDH | [33] |
| 68 |  | MM | V=3.04mM/min, kcat=57760,  Km=0.06mM | , | LDH | [33] |
| 69 |  | MM | V=11.7mM/min, kcat=386100,  Km=150mM |  | MCT2 | [33] |
| 70 |  | MM | V=11.7mM/min, kcat=222300,  Km=150mM |  | MCT1 | [33] |
| 71 |  | HMM | V=803000000mM/min,Km=0.2mM |  |  | [33] |
| 72 |  | HMM | V=0.2774mM/min,Km=0.2mM |  |  | [33] |
| 73 |  | HMM | V=16.72mM/min,Km=47.2mM | ,, |  | [33] |
| 74 |  | HMM | V=29.04mM/min,Km=47.2mM | ,, |  | [33] |
| 75 |  | HMM | V=16.72mM/min,Km=20mM | , |  | [33] |
| 76 |  | HMM | V=16.72mM/min,Km=20mM | , |  | [33] |
| 77 |  | HMM | V=1.66x10^12^mM/min,Km=0.00014mM |  |  | [33] |
| 78 |  | HMM | V=0.0703mM/min,Km=0.00014mM |  |  | [33] |
| 79 |  | HMM | V=2755mM/min,Km=0.00019mM |  |  | [33] |
| 80 |  | HMM | V=0.703mM/min,Km=0.00019mM |  |  | [33] |
| 81 |  | HMM | V=250868.37mM/min,Km=10.14mM |  |  | [33] |
| 82 |  | HMM | V=0.703mM/min,Km=10.14mM |  |  | [33] |
| 83 |  | HMM | V=2366493.65mM/min,Km=0.51mM |  |  | [33] |
| 84 |  | HMM | V=0.703mM/min,Km=0.51mM |  |  | [33] |
| 85 |  | HMM | V=0.0447mM/min,Km=0.099mM |  |  | [33] |
| 86 |  | HMM | V=0.703mM/min,Km=0.099mM |  |  | [33] |
| 87 |  | HMM | V=1.2654mM/min,Km=1.74mM |  |  | [33] |
| 88 |  | HMM | V=0.703mM/min,Km=1.74mM |  |  | [33] |
| 89 |  | HMM | V=74.76mM/min,Km=2.82mM |  |  | [33] |
| 90 |  | HMM | V=0.703mM/min,Km=2.82mM |  |  | [33] |
| 91 |  | HMM | V=0.18mM/min,Km=13.48mM |  |  | [33] |
| 92 |  | HMM | V=4.67mM/min,Km=13.48mM |  |  | [33] |
| 93 |  | MM | kcat=19000min^-1^ ,Km=0.27mM |  | MCT | [33] |
| 94 |  | MM | kcat=71610min^-1^ ,Km=8.13mM |  | LDH | [33] |
